# Long-Term Outcomes of Interventional Bronchoscopy for Central Airway Obstruction in a Single-Center Retrospective Study: A Subgroup Analysis of Malignant and Benign Lesions †

**DOI:** 10.3390/jcm14072155

**Published:** 2025-03-21

**Authors:** Paolo Scanagatta, Francesco Inzirillo, Giuseppe Naldi, Casimiro Eugenio Giorgetta, Eugenio Ravalli, Gianluca Ancona, Sara Cagnetti

**Affiliations:** Division of Thoracic Surgery, Morelli Hospital, ASST Valtellina e Alto Lario, 23035 Sondalo (SO), Italy; francesco.inzirillo@asst-val.it (F.I.); giuseppe.naldi@asst-val.it (G.N.); casimiro.giorgetta@asst-val.it (C.E.G.); eugenio.ravalli@asst-val.it (E.R.); gianluca.ancona@asst-val.it (G.A.); sara.cagnetti@asst-val.it (S.C.)

**Keywords:** rigid bronchoscopy, lung cancer, central airway obstruction, palliation, thoracic malignancies, stent placement, multimodal therapy

## Abstract

**Background:** Central airway obstruction (CAO) remains a major challenge in thoracic oncology, particularly in patients with advanced lung cancer. Despite advances in systemic therapies, interventional bronchoscopy, especially rigid bronchoscopy (RB), plays a critical role in managing CAO. **Methods:** Between June 2005 and December 2023, 416 patients with central airway obstructions were treated. The cohort included 213 males (51%) and 203 females (49%), with a mean age of 64.8 years. A retrospective review of patient data was conducted, and descriptive statistics were used to summarize demographics and procedural outcomes. Survival rates and complication data were analyzed using Kaplan–Meier survival curves. Multivariate analysis was performed to identify significant predictors of survival and complications, considering factors such as tumor stage, prior treatments, and comorbidities. The study also analyzed the impact of adjuvant therapies and stenting on patient outcomes. **Results:** Of the 416 patients, 86 (20.7%) had benign lesions, including 61 cases of post-tracheostomy stenosis or prolonged intubation, while the remaining 330 (79.3%) had malignant CAO. Patients receiving adjuvant therapies showed a significant survival advantage, with a median survival of 24 months compared to 15 months for those not receiving adjuvant therapies (*p* = 0.015). Stenting was performed in 141 cases, but no significant survival difference was found between patients with and without stents (*p* = 0.52). Complications were rare, with airway rupture observed in 1.9% and perioperative mortality in 0.25%. Symptom relief was achieved in the majority of patients, with significant improvements in quality of life, particularly in those with malignant obstructions. **Conclusions:** RB remains a cornerstone in the management of CAO, providing effective and durable symptom relief. It is particularly beneficial for advanced malignancies, offering a palliative approach that improves both survival and quality of life. Our study supports the guidelines endorsing RB for CAO management and highlights its role in providing significant symptom relief and stabilization in patients with severe airway obstruction.

## 1. Introduction

Continuous advancements in chemotherapy and radiotherapy have led to increased survival rates among patients with lung cancer. However, despite these innovations, malignant central airway obstruction (CAO) remains a significant clinical challenge, affecting approximately 20–30% of individuals with primary lung cancer during their disease course. This condition often leads to severe respiratory distress, necessitating urgent interventions to alleviate symptoms and improve quality of life [1].

Malignant CAO frequently involves the trachea and main bronchi, resulting in significant morbidity and mortality. Traditional systemic therapies, such as chemotherapy and radiotherapy, rarely succeed in resolving airway obstructions or managing related complications. Consequently, interventional bronchoscopy, particularly rigid bronchoscopy (RB), has become a cornerstone in the palliative management of advanced-stage thoracic malignancies. The advent of advanced bronchoscopic techniques, including laser ablation, argon plasma coagulation, cryotherapy, and the placement of airway stents, has further expanded the therapeutic options available for managing CAO [2,3]

Rigid bronchoscopy offers distinct advantages over flexible bronchoscopy, particularly in cases of severe CAO. It provides superior airway stabilization, facilitates the use of multiple instruments, and ensures effective bleeding control through tamponade. These capabilities make RB indispensable for mechanically debulking airway lesions, dilating stenotic airways, and placing self-expanding metallic or silicone stents [4]. Studies have shown that combining rigid bronchoscopic techniques with multimodal therapies can lead to significant symptomatic relief, reduced dyspnea scores, and improved quality of life in the majority of patients [5].

The importance of interventional bronchoscopy in thoracic oncology is further highlighted by its ability to serve as a complementary modality to systemic treatments. While systemic therapies target tumor progression, bronchoscopic interventions aim to restore airway patency, alleviate respiratory distress, and provide immediate symptomatic relief, especially in patients with advanced-stage lung cancer who are unresponsive to other therapies [6,7].

This study reviews our 18-year experience with interventional bronchoscopy in patients presenting with malignant CAO. We focus on the safety and efficacy of various rigid bronchoscopic techniques in achieving airway patency, managing life-threatening complications, and improving patient outcomes. By contextualizing our findings within the broader scope of thoracic oncology, we aim to underscore the critical role of interventional bronchoscopy in modern multidisciplinary cancer care [8].

## 2. Materials and Methods

### 2.1. Study Design and Setting

This retrospective study was conducted at the Thoracic Endoscopy Unit of Morelli Hospital, ASST Valtellina e Alto Lario, Sondalo, Italy. Data were collected from June 2005 to December 2023, encompassing all patients undergoing interventional bronchoscopy for central airway obstruction (CAO).

### 2.2. Patient Selection

A total of 416 consecutive patients presenting with either benign or malignant central airway obstructions were included. Patients were categorized based on the etiology of the obstruction: 330 (79.3%) with malignant obstructions and 86 (20.7%) with benign lesions. Benign conditions included post-tracheostomy or prolonged intubation stenosis, tracheomalacia, foreign body obstruction, tracheobronchial amyloidosis, and bronchopleural fistulas. Malignant obstructions involved tumors affecting the trachea (proximal, mid, or distal), main bronchi, bronchus intermedius, and lobar bronchi.

### 2.3. Procedure and Techniques

All bronchoscopic procedures were performed using rigid bronchoscopes under general anesthesia. Interventions included mechanical and laser ablation, balloon dilation, stent placement, and stent removal. A total of 372 therapeutic procedures were conducted for malignant obstructions, with some patients requiring multiple interventions to maintain airway patency.

Key procedural techniques included:

- Mechanical and Laser Ablation: Performed in 301 procedures to restore airway patency [5].
- Balloon Dilation: Combined with other techniques in 44 procedures.
- Stent Placement and Removal: Stents were placed in 141 procedures to maintain airway patency, using both metallic and silicone stents depending on the clinical indication [2].

### 2.4. Data Collection and Outcome Measures

Data were retrospectively reviewed from patient medical records, including demographic details, procedural indications, and clinical outcomes. Procedural success was defined as achieving at least 50% luminal patency, as assessed by direct bronchoscopy and clinical symptom improvement. Complications, such as airway rupture, bleeding, or death, were recorded and categorized by severity.

#### Assessment of Improvement

Subjective improvement in respiratory symptoms and quality of life was evaluated using a 5-point scale, structured as follows: 1 = significant worsening, 2 = slight worsening, 3 = no change, 4 = slight improvement, 5 = significant improvement. This assessment was conducted using one or more of the following methods, depending on data availability for each patient:

- Retrospective review of medical records, including notes from surgical, pulmonology, or oncology outpatient visits.
- Telephone contact at 30 days post-procedure, either with the patient or, if not feasible, with the primary caregiver.
- Analysis of any completed EORTC QLQ-C30 and QLQ-LC13 questionnaires during post-procedural follow-up.
- Documented reduction in the need for or dosage of oxygen therapy in oxygen-dependent patients.

All patients were included in this analysis, except the patient who died. The integration of these methods allowed for a comprehensive evaluation, combining both subjective and objective data to provide a more accurate analysis of the clinical impact of the procedure.

**Figure 1:**
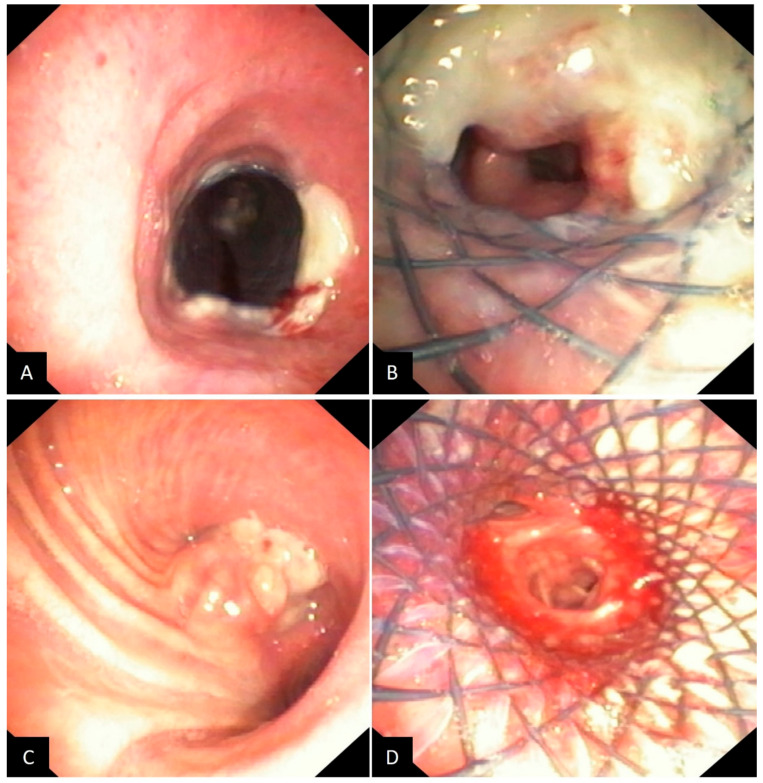
(**A**) Tracheal obstruction removal with stent (proximal view); (**B**) Same case (distal view); (**C**) Complete left main bronchus obstruction at the origin; (**D**) Same case after disobstruction and stent placement (distal view).

**Figure 2:**
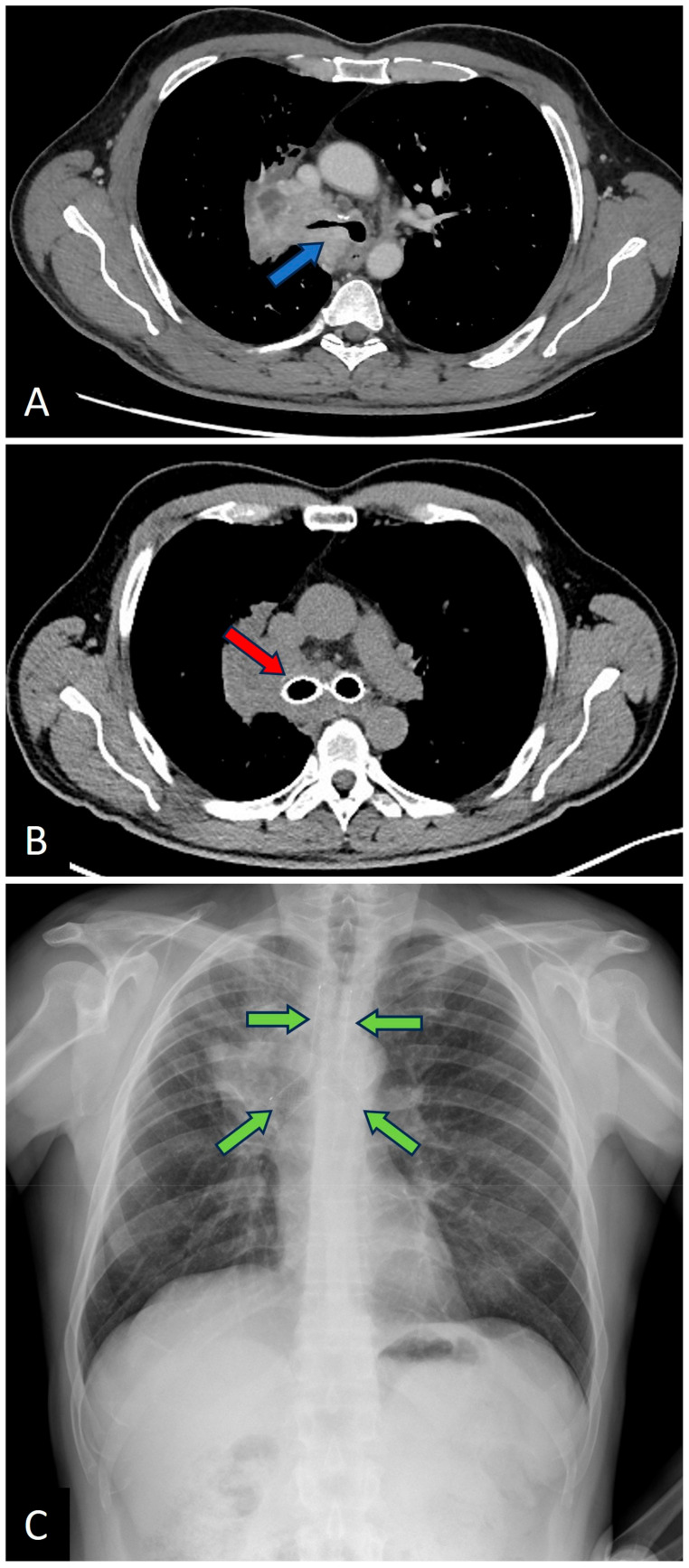
(**A**) Chest CT showing obstruction of the right main bronchus and carina (blue arrow); (**B**) Follow-up chest CT after Y-stent placement (red arrow); (**C**) Chest X-ray showing the same case (stent clearly visible, green arrows).

For illustration of the procedural outcomes, Figure 1 and Figure 2 show images of airway obstructions and the resulting therapeutic interventions performed on representative cases.

### 2.5. Statistical Analysis

Descriptive statistics were used to summarize patient demographics, procedural outcomes, and clinical variables. Categorical data were presented as frequencies and percentages, while continuous variables were expressed as means with standard deviations for normally distributed data or medians with interquartile ranges for non-normally distributed data.

To assess differences between groups, the following statistical tests were used:

- Chi-square test or Fisher’s exact test for categorical variables to compare the distribution of patient characteristics across subgroups (e.g., adjuvant therapies vs. no adjuvant therapies, stenting vs. no stenting).
- Independent *t*-test for continuous variables (e.g., age, survival time) between two groups when the data were normally distributed. If the data were not normally distributed, the Mann–Whitney U test was used.
- Log-rank test was performed to compare survival curves between different subgroups (e.g., adjuvant therapy vs. no adjuvant therapy, stent vs. no stent). Survival analysis was conducted using the Kaplan–Meier method.
- Multivariate Cox proportional hazards regression analysis was used to adjust for confounding factors, such as tumor location, prior treatments, and comorbidities, and to identify independent predictors of survival.

Subgroup analyses were performed for patients based on tumor location, prior treatments (e.g., chemotherapy, radiotherapy), and comorbidities (e.g., diabetes, cardiovascular disease). Statistically significant differences were determined using a *p*-value threshold of <0.05. All statistical tests were two-sided, and the statistical significance was considered at the 5% level.

#### Exclusion of Early Deaths

Patients who died within 30 days of treatment were excluded from the survival analysis. This decision was made to avoid the influence of early deaths, which could be related to pre-existing comorbidities or immediate complications unrelated to treatment efficacy. Excluding these patients is a common practice in survival analyses, as it allows for a more accurate assessment of the long-term impact of treatment, focusing on deaths that are more likely related to the disease progression or the effectiveness of the interventions.

## 3. Results

### 3.1. Patient Demographics and Characteristics

A total of 416 patients (213 males, 51%, and 203 females, 48%) with malignant central airway obstructions were treated between June 2005 and December 2023. The patients’ ages ranged from 22 to 86 years, with a mean age of 64.8 years (see Table 1 for patient demographics and characteristics).

### 3.2. Distribution of Airway Obstructions

The majority of patients (over 80%) presented with severe airway obstruction (>75% luminal narrowing), while a smaller proportion (approximately 20%) had moderate obstructions (50–75% narrowing). Such a high prevalence of severe obstructions underscores the need for effective therapeutic interventions, particularly in patients with advanced-stage malignancies where rapid symptom relief and airway stabilization are crucial.

The obstructions predominantly affected the following anatomical sites:

- Trachea or carina: 159 patients
- Left main bronchus: 70 patients
- Right main bronchus: 61 patients
- Bronchus intermedius: 49patients
- Lobar bronchi: 77 patients

### 3.3. Therapeutic Procedures

A total of 514 interventional bronchoscopies were performed, utilizing both rigid and flexible techniques. Rigid bronchoscopies were conducted in 372 cases (301 with laser and mechanical ablation, 44 with balloon dilation, and 27 for stent removal only).

The interventions included:

- Laser and Mechanical Ablation: 301 procedures aimed at restoring airway patency (all rigid bronchoscopies).
- Balloon Dilations: Performed in 44 procedures (rigid bronchoscopy).
- Stent Placements: Conducted in 141 procedures, with 179 stents deployed in total (111 single stents and 30 multiple stents). The stents used included:
  o Tracheal metallic: 35
  o Tracheal silicone: 30
  o Bronchial metallic: 39
  o Bronchial silicone: 34
  o Y-shaped stents: 41

These data are summarized in Table 2.

### 3.4. Results for Benign Lesions

Among the 416 patients, 86 (20.7%) had benign airway lesions, including:

- 61 cases of post-tracheostomy stenosis or post-prolonged intubation
- 5 cases of foreign body obstruction
- 5 cases of tracheomalacia (one of which due to Mounier-Kuhn Syndrome)
- 4 cases of tracheobronchial amyloidosis
- 11 cases of bronchopleural fistulas

The distribution of airway obstructions in benign lesions was as follows:

- Trachea or Carina: 61 patients (70.9%)
- Left Main Bronchus: 3 patients (3.5%)
- Right Main Bronchus: 7 patients (8.1%)
- Bronchus Intermedius: 6 patients (7%)
- Lobar Bronchi: 9 patients (10.5%)

Compared to patients with malignant lesions, benign lesions were more frequently localized to the trachea or carina, often secondary to prolonged intubation or tracheostomy.

### 3.5. Outcomes

The overall outcomes were assessed in terms of symptom relief, survival, and complications.

- Symptom Relief: Symptom relief was assessed using a 5-point scale, with 1 representing significant worsening and 5 representing significant improvement. A combination of retrospective medical record review, telephone follow-up, and completed quality-of-life questionnaires (EORTC QLQ-C30 and QLQ-LC13) were used to gather data. Additionally, for oxygen-dependent patients, documented reduction in oxygen therapy use was considered as an objective measure of symptom improvement.

The majority of patients (80%) experienced significant to slight improvement in their symptoms after the intervention. These improvements were reflected in the reduction in dyspnea, cough, and wheezing, which are common symptoms in patients with central airway obstruction (CAO). Specifically, 77.5% of patients receiving adjuvant therapies reported significant or slight improvement, while 82.5% of patients in the no adjuvant therapy group reported similar relief. Conversely, 20% of patients either showed no change or had worsening symptoms, though this was more common in the no-adjuvant therapy group.

These results were consistent across different techniques, including rigid bronchoscopy with laser and mechanical ablation, balloon dilation, and stent placement, all of which were effective in improving airway patency and reducing respiratory distress. Symptom relief was most notable in patients with severe airway obstructions, particularly in those with advanced-stage malignancies who had not responded to systemic treatments.

The data regarding symptom relief are detailed in Table 3, where the proportion of patients showing significant improvement, no change, or worsening of symptoms are clearly presented. Importantly, there was no significant difference in symptom relief between patients who received adjuvant therapies and those who did not, suggesting that the interventions themselves, rather than the addition of adjuvant therapies, were the primary contributors to the observed improvement.

**Figure 3:**
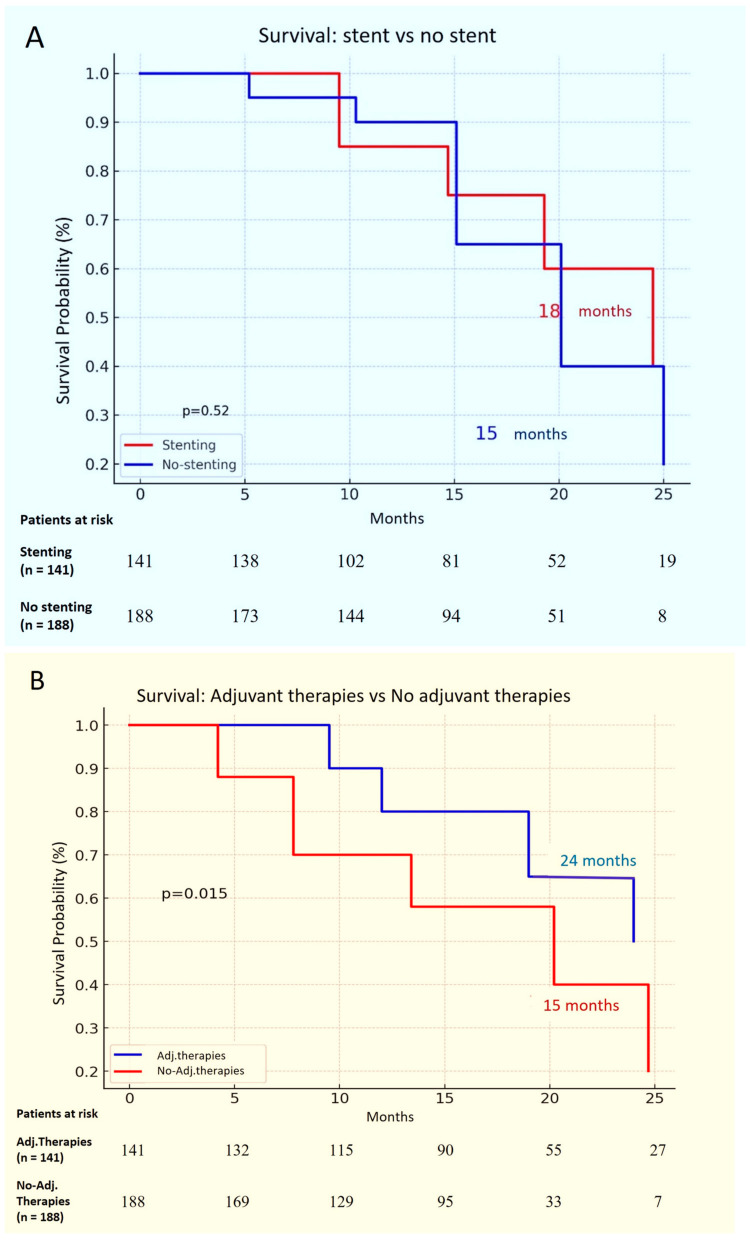
(**A**) First Survival Curve: *Survival: Stenting vs. No Stenting* Kaplan–Meier survival curve comparing patients who received stenting (red line) versus those who did not (blue line). The survival probability is plotted against time in months. The median survival for patients with stenting was 18 months, while for those without stenting, it was 15 months. The *p*-value is 0.52, indicating no significant difference in survival between the two groups. (**B**) Second Survival Curve: *Survival: Adjuvant Therapies vs. No Adjuvant Therapies* Kaplan–Meier survival curve comparing patients who received adjuvant therapies (blue line) versus those who did not (red line). The survival probability is plotted against time in months. Patients receiving adjuvant therapies had a median survival of 24 months, while those not receiving adjuvant therapies had a median survival of 15 months. The *p*-value is 0.015, suggesting a statistically significant difference in survival between the two groups.

- Long-term Follow-up: During the study, a subset of patients required multiple bronchoscopic interventions to maintain airway patency. Specifically, 64 patients (15.4%) underwent more than one procedure, typically due to persistent obstruction or re-obstruction. While the majority of patients did not experience significant long-term complications, repeat interventions were needed in cases where airway patency could not be maintained with a single procedure. This indicates the ongoing need for monitoring and possible re-treatment, especially in patients with complex or recurrent central airway obstructions.
- Survival: The median survival time was 16.4 months, with a range from 1 to 31 months. Subgroup analyses revealed that patients who received adjuvant therapies had significantly improved survival rates compared to those who did not (log-rank *p* = 0.015). The survival curves for adjuvant therapies versus no adjuvant therapies are depicted in Figure 3A,B. However, no other statistically significant associations were found for survival based on other factors.

- Severity of Airway Obstruction: the majority of patients had severe airway obstruction (>75% narrowing) (80%), while the remaining 20% had moderate obstructions (50–75% narrowing). This distribution emphasizes the need for effective therapeutic interventions, particularly for patients with advanced-stage malignancies where airway stabilization is critical.
- Complications: As illustrated in Table 3 The overall complication rate was low:
  o Airway Ruptures: These occurred in 8 patients (1.9%) but were managed without septic sequelae.
  o Massive Hemorrhage and Perioperative Death: This occurred in 1 patient (0.25%), highlighting the risk, although rare, of major bleeding during the procedure.
- Subgroup Analysis: In 28 patients, interventions were required due to persistent major airway obstruction caused by small cell lung cancer (SCLC) despite chemotherapy. In these cases, rigid bronchoscopy provided effective palliation and symptom control.
- Multivariate Analysis: The multivariate analysis demonstrated a significant increase in the risk of complications and mortality for patients with stage IV disease, with a median overall survival of 6.2 months. For patients with prior chemotherapy or radiotherapy, the median survival was 7.4 months. Severe cardiovascular comorbidities were also significantly associated with an increased risk of complications and mortality, with an odds ratio (OR) of 2.2, a 95% confidence interval (CI) of 1.4–3.3, and a *p*-value < 0.01. Specifically, for stage IV disease, the OR was 3.4 (CI: 2.2–5.2, *p* < 0.001), and for those with prior chemotherapy or radiotherapy, the OR was 2.3 (CI: 1.5–3.5, *p* < 0.01). These results indicate that advanced disease stage, previous treatments, and severe cardiovascular comorbidities are important independent predictors of poor outcomes in this cohort.

Note:

- Median Survival for the benign lesions group was not reached, indicating that survival data could not be accurately measured within the time frame of the study.
- Symptom Improvement (QoL) shows no significant difference between the groups, with the benign lesions group showing 79% improvement, comparable to the treatment groups.
- Complications: Severe complications were absent in the benign group (*p* = 0.01), while mild complications (such as controlled bleeding, infections, arrhythmias, and hospitalization > 3 days) affected 25.5% of benign cases, but there were no significant differences across the groups.

## 4. Discussion

The management of central airway obstruction (CAO) has evolved significantly, with recent clinical practice guidelines from the American College of Chest Physicians (ACCP), published in 2024 by Mahmood et al., reinforcing the critical role of rigid bronchoscopy in the treatment of malignant CAO [9]. These guidelines emphasize a multimodal approach, incorporating interventional techniques such as rigid bronchoscopy, laser ablation, and stent placement to ensure rapid restoration of airway patency and improve patient quality of life [10]. Our study aligns with these recommendations, confirming the effectiveness and safety of rigid bronchoscopy as a palliative treatment for patients with central airway obstruction caused by advanced thoracic malignancies.

Untreated central airway obstruction (CAO) in patients with lung cancer leads to a rapid deterioration in prognosis, with significant morbidity and a generally poor short-term survival rate. According to Daneshvar et al. (2019), the prevalence and outcomes of CAO in lung cancer patients underscore the critical need for timely and effective intervention. Without proper management, CAO can severely compromise respiratory function and quality of life, leading to rapid decline and higher mortality rates [11].

The present study highlights the pivotal role of rigid bronchoscopy (RB) in the management of malignant central airway obstruction (CAO), a condition associated with significant morbidity and mortality among patients with thoracic malignancies. Over an 18-year period, 416 patients with CAO underwent 514 interventional bronchoscopic procedures, achieving substantial symptom relief in most cases. This procedural volume and success rate underscore the critical role of RB in addressing CAO. A closer analysis of the anatomical sites involved provides further insight into the challenges posed by these cases. The most frequent central anatomical sites of obstruction were the distal trachea or carina, the left main bronchus, and the right main bronchus.

These locations are pivotal for maintaining airway patency, making the low complication rates observed in this study particularly notable. Among these patients, laser and mechanical ablations were the most commonly performed interventions (301 procedures), while stent placements were required in 141 cases to ensure long-term airway patency. Notably, the complication rate was low, with airway ruptures observed in 1.9% of cases and perioperative mortality in only 0.25% of cases. Such outcomes are consistent with findings in the literature, further validating RB’s safety and efficacy as a palliative intervention [7].

These findings underscore the effectiveness and safety of RB as a palliative modality in thoracic oncology, offering immediate and durable symptom relief even in advanced-stage lung cancer patients who are refractory to systemic therapies. The results align with existing literature, which consistently supports the use of RB in restoring airway patency and improving the quality of life for patients with obstructive airway malignancies [2].

The findings of this study are consistent with those reported in the existing literature on the use of rigid bronchoscopy (RB) for malignant central airway obstruction (CAO). These parallels underscore the importance of multimodal approaches, which combine various techniques to enhance outcomes. Previous studies have demonstrated that RB offers distinct advantages in managing airway obstructions, particularly in cases requiring immediate and effective palliation. For instance, Khan et al. (2020) reported similar success rates in restoring airway patency and achieving symptom relief through multimodal RB interventions, including mechanical debulking, balloon dilation, and stent placement [10]. Similarly, Criner et al. (2020) emphasized the importance of combining techniques such as laser ablation and stenting to optimize therapeutic outcomes in patients with thoracic malignancies [6].

The low complication rates observed in our study also align with the findings of Khan et al., who reported minimal perioperative morbidity and mortality associated with RB [10]. The rate of airway rupture in our cohort (1.9%) was comparable to or lower than rates reported in other series, highlighting the safety of the procedure when performed in specialized centers. The perioperative mortality rate in our study (0.25%) is among the lowest documented, further underscoring the efficacy and safety of RB as a palliative intervention [10].

A notable contribution of our study is the high rate of immediate symptom relief, achieved in nearly all patients following RB interventions. This outcome is consistent with the work of Huret et al. (2015), who reported significant improvements in dyspnea scores and overall respiratory function following RB procedures [12]. The median survival of 16.4 months observed in our cohort is also within the range reported in similar studies, underscoring the role of RB not only as a palliative measure but also as a means to stabilize patients and extend their survival in certain clinical scenarios.

Compared to flexible bronchoscopy (FB), RB offers several key advantages, as highlighted in both this study and the literature. FB, while effective for diagnostic purposes, often falls short in managing severe obstructions or achieving long-lasting results. The large working channel of the rigid bronchoscope allows for the deployment of advanced tools, such as stents and lasers, which are critical for addressing complex airway lesions. This ability to perform comprehensive therapeutic interventions during a single session distinguishes RB as a cornerstone of interventional pulmonology [12].

Rigid bronchoscopy (RB) is uniquely positioned as a critical modality in the management of central airway obstruction (CAO), particularly in cases of malignant obstruction where rapid symptom relief and airway stabilization are paramount. Unlike flexible bronchoscopy (FB), RB allows for simultaneous deployment of multiple therapeutic tools, providing the operator with greater control during complex procedures. This advantage is particularly evident in the management of severe obstructions or in emergency settings where immediate restoration of airway patency is required.

One of the defining features of RB is its ability to manage life-threatening complications, such as massive bleeding or airway rupture, with superior efficiency compared to FB. The rigid bronchoscope’s design ensures sustained ventilation during procedures, a critical factor in maintaining oxygenation while performing intricate interventions. Additionally, the ability to apply tamponade directly to bleeding sites makes RB an indispensable tool for managing perioperative hemorrhages [2].

RB also facilitates the placement of stents, which are essential for maintaining airway patency following ablative procedures. In our cohort, 141 procedures involved stent placement, with both metallic and silicone stents effectively used to address a variety of anatomical challenges. The literature supports the use of stents as a reliable method to achieve long-term symptom relief, with studies such as those by Huret et al. (2015) and Khan et al. (2020) highlighting similar outcomes [10,12]. The versatility of RB in accommodating stents of various types and configurations, including Y-shaped stents for complex lesions, further emphasizes its value in thoracic oncology.

In emergency settings, RB plays a pivotal role in rapidly restoring airway patency, often serving as the first-line intervention for CAO. This capability is particularly important in patients presenting with critical airway narrowing or respiratory distress due to tumor progression. Studies, including Criner et al. (2020), have highlighted the importance of prompt bronchoscopic intervention in improving survival and quality of life in such scenarios [6].

Despite its advantages, RB requires specialized expertise and infrastructure, including operating room facilities and anesthesia support. These requirements limit its accessibility in some settings but also underscore the importance of concentrating such interventions in high-volume centers where outcomes can be optimized. As demonstrated in our study, the outcomes of RB are significantly influenced by operator experience and institutional resources.

The management of malignant central airway obstruction (CAO) requires a comprehensive and multimodal approach, as no single technique can address the diverse challenges posed by complex airway lesions. In our study, the combination of laser and mechanical ablation, balloon dilation, and stent placement proved effective in achieving immediate symptom relief and maintaining airway patency over time. This aligns with the growing consensus in the literature that multimodal treatment strategies are essential for optimizing outcomes in patients with CAO [7].

### 4.1. Study Limitations

While this study provides valuable insights into the role of rigid bronchoscopy (RB) in managing malignant central airway obstruction (CAO), several limitations should be acknowledged:

1. Retrospective Design

Being a retrospective analysis, the study relies on previously recorded data, which may introduce selection and reporting biases. Prospective, randomized trials would provide stronger evidence for the effectiveness and safety of RB interventions [9].

2. Lack of a Control Group

The absence of a comparative cohort limits the ability to assess the relative efficacy of RB versus other modalities, such as flexible bronchoscopy (FB) or non-bronchoscopic interventions (e.g., radiotherapy or chemotherapy alone).

3. Variability in Techniques and Operators

Procedures were performed over an 18-year period, during which advancements in technology and variations in operator expertise may have influenced outcomes. Standardizing techniques and comparing outcomes across different operators and time periods would offer a clearer understanding of the intervention’s impact.

4. Focus on Malignant CAO

While the study primarily addresses malignant obstructions, a broader analysis including benign conditions could provide a more comprehensive overview of RB’s versatility and applications [4,13].

5. Limited Long-Term Follow-Up Data

Although the median survival was reported, detailed data on long-term quality of life, stent durability, and late complications (e.g., granulation tissue formation) were not extensively analyzed [14].

### 4.2. Suggestions for Future Studies

To address these limitations and further refine the role of RB in managing CAO, future research should focus on:

- Prospective and Randomized Studies

Comparing RB to alternative or combined modalities, including FB and systemic therapies, to identify the most effective and cost-efficient approaches [7].

- Quality-of-Life Assessments

Incorporating patient-reported outcomes and validated quality-of-life measures to better understand the palliative benefits of RB [6].

- Cost-Effectiveness Analysis

Assessing the economic impact of RB interventions relative to other therapeutic strategies, particularly in resource-limited settings.

- Innovative Technologies

Exploring the use of emerging technologies, such as robotic bronchoscopy and advanced stent materials, to enhance procedural safety and outcomes.

- Long-Term Follow-Up Monitoring

Investigating the long-term impact of RB on airway patency, symptom control, and survival, particularly in patients with chronic stent placement [9].

### 4.3. Clinical Implications

Rigid bronchoscopy (RB) represents an essential tool in the armamentarium of thoracic oncology, particularly for the management of malignant central airway obstruction (CAO). The results of this study reaffirm RB’s critical role as a palliative procedure capable of offering rapid symptom relief, restoring airway patency, and stabilizing patients for further oncologic treatments.

1. Integration into Multidisciplinary Care

The integration of interventional bronchoscopy into multidisciplinary thoracic oncology teams ensures timely and effective management of CAO, especially in patients unresponsive to systemic therapies. RB not only complements chemotherapy and radiotherapy but also enhances their efficacy by improving respiratory function and reducing complications such as recurrent infections or hypoxia [2].

2. Impact on Quality of Life

The ability of RB to provide immediate relief from symptoms such as dyspnea significantly improves the quality of life for patients in advanced stages of lung cancer. Moreover, the use of stents and multimodal techniques ensures sustained airway patency, reducing the need for repeated hospitalizations and interventions. This is particularly critical in the palliative care setting, where preserving dignity and comfort is paramount [7].

3. Management of Emergency CAO

RB’s capability to rapidly address life-threatening obstructions makes it an indispensable intervention in emergency settings. For patients presenting with severe respiratory distress or imminent airway compromise, RB offers a potentially life-saving option [6].

4. Education and Training

Given the complexity and potential risks associated with RB, there is a pressing need to expand training programs and improve accessibility to interventional pulmonology expertise. High-volume centers like ours play a vital role in ensuring the availability of experienced operators and the latest technologies [14].

### 4.4. Future Perspectives

1. Technological Advancements

The development of robotic-assisted bronchoscopy and novel stent designs holds promise for improving the precision, safety, and long-term outcomes of RB. These innovations may further expand the indications for RB and reduce procedural risks [9].

2. Standardization of Protocols

Establishing standardized protocols for the use of RB in CAO, including guidelines for multimodal interventions and stent selection, would help ensure consistent outcomes across different centers [6,9].

3. Focus on Personalized Care

Advances in imaging and biomarker research could enable more personalized approaches to CAO management. Tailoring interventions based on tumor characteristics, anatomical variations, and patient-specific factors may optimize results and minimize complications. The ability to personalize treatment plans will be crucial to improving long-term outcomes and reducing the likelihood of complications, as highlighted by recent studies [7,8,9].

4. Expanding Access to Care

Increasing the availability of interventional bronchoscopy in resource-limited settings remains a significant challenge. Future efforts should focus on training more operators, equipping centers with the necessary infrastructure, and developing cost-effective solutions to expand access to this life-saving modality [9].

## 5. Conclusions

Rigid bronchoscopy (RB) is a key modality in the management of central airway obstruction (CAO), providing significant benefits in symptom relief, airway stabilization, and quality-of-life improvement across both malignant and benign conditions. Over 18 years, our study confirms RB’s safety and efficacy in delivering durable outcomes, even in advanced-stage disease and patients with severe comorbidities.

RB, when integrated into multidisciplinary thoracic oncology teams, complements systemic therapies and allows for effective management of CAO. The use of multimodal techniques, including laser ablation, mechanical debulking, balloon dilation, and stent placement, further underscores its versatility.

In conclusion, expanding access to RB, refining protocols, and incorporating emerging technologies could maximize its impact in improving both survival and quality of life for patients with CAO.

## Figures and Tables

**Table 1:** Patient demographics, airway obstruction characteristics, and tumor information.

Variable	Total (n = 416)	Benign Lesions (n = 86)	Adjuvant Therapies (n = 141)	No Adjuvant Therapies (n = 189)
**Age**				
Mean (SD)	64.8 (± 12.5)	61.9 (± 12.8)	64.1 (± 12.4)	65.4 (± 12.8)
**Gender**				
Male (%)	213 (51.2%)	44 (51.2%)	72 (51.1%)	107 (56.6%)
Female (%)	203 (48.8%)	42 (48.8%)	69 (48.9%)	82 (43.4%)
**Comorbidity**				
**- Cardiological**				
Hypertension (%)	132 (31.7%)	26 (30.2%)	44 (31.2%)	62 (32.8%)
Severe cardiovascular diseases (%)	60 (14.4%)	14 (16.3%)	20 (14.2%)	26 (13.8%)
**- Metabolic**				
Type II Diabetes (%)	108 (26%)	24 (27.9%)	34 (24.1%)	50 (26.5%)
**Distribution of Airway Obstruction**				
Trachea or Carina	159	61	42	56
Left Main Bronchus	70	3	28	39
Right Main Bronchus	61	7	22	32
Bronchus Intermedius	49	6	19	24
Lobar Bronchi	77	9	30	38
**Benign Lesions**	86 (20.7%)			
**Malignant Lesions**	330 (79.3%)		141	189
**Tumor Type**				
Squamous cell carcinoma (%)	172 (52%)	-	64 (45.7%)	108 (57.1%)
Adenocarcinoma (%)	115 (35%)	-	51 (36.2%)	64 (33.9%)
Small cell carcinoma (%)	28 (8.5%)	-	12 (8.5%)	16 (8.5%)
Large cell carcinoma (%)	15 (4.5%)	-	9 (6.4%)	6 (3.2%)
**Stage (TNM)**				
Stage III (%)	248 (75.2%)	-	109 (77.3%)	139 (73.5%)
Stage IV (%)	82 (24.8%)	-	32 (22.7%)	50 (26.5%)

**Table 2:** Breakdown of therapeutic procedures for malignant airway obstructions.

Procedure Type	Number of Procedures
Laser and Mechanical Ablation	301
Balloon Dilation	53
Stent Placement	150
Total Stents Deployed	179
- Tracheal Metallic	35
- Tracheal Silicone	30
- Bronchial Metallic	39
- Bronchial Silicone	34
- Y-shaped Stents	41

**Table 3:** Morbidity, mortality, and quality of life by subgroup.

Variable	Malignant Obstructions (n = 330)	Benign Lesions (n = 86)	Adjuvant Therapies (n = 141)	No Adjuvant Therapies (n = 189)	*p*-Value
**Median Survival (months)**	16.4	Not reached	24.0	15.0	0.015
**Symptom Improvement (QoL)**					
Significant + Slight Improvement (%)	264 (80%)	68 (79%)	109 (77.5%)	155 (82.5%)	NS
No Change + Worsening or No Response (%)	66 (20%)	18 (21%)	32 (22.5%)	34 (17.5%)	NS
**Complications**					
**Severe Complications**		0 (0%)	3 (2.1%)	5 (2.6%)	**0.01**
Airway Ruptures (%)	8 (1.9%)	0 (0%)	3 (2.1%)	5 (2.6%)	NS
Massive Hemorrhage and Perioperative Death (%)	1 (0.25%)	0 (0%)	0 (0%)	1 (0.5%)	NS
**Mild Complications**		22 (25.5%)	26 (18.4%)	31 (16.4%)	NS

## Data Availability

The data presented in this study are available on request from the corresponding author.
